# Gamma-ray vortices from nonlinear inverse Thomson scattering of circularly polarized light

**DOI:** 10.1038/s41598-017-05187-2

**Published:** 2017-07-10

**Authors:** Yoshitaka Taira, Takehito Hayakawa, Masahiro Katoh

**Affiliations:** 1 Research Institute for Measurement and Analytical Instrumentation, National Metrology Institute of Japan, National Institute of Advanced Industrial Science and Technology (AIST), Tsukuba Central 2, 1-1-1 Umezono, Tsukuba, Ibaraki 305-8568 Japan; 2 Department of Physics and Astronomy, Mississippi State University, 355 Lee Blvd., 125 Hilbun Hall, Mississippi State, Mississippi 39762 USA; 3 National Institutes for Quantum and Radiological Science and Technology, Tokai, Ibaraki 319-1106 Japan; 4 National Astronomical Observatory of Japan, Mitaka, Tokyo 181-8588 Japan; 5 Institute for Molecular Science, National Institutes of Natural Sciences/School of Physical Sciences, SOKENDAI (The Graduate University for Advanced Studies), Okazaki, Aichi 444-8585 Japan

## Abstract

Inverse Thomson scattering is a well-known radiation process that produces high-energy photons both in nature and in the laboratory. Nonlinear inverse Thomson scattering occurring inside an intense light field is a process which generates higher harmonic photons. In this paper, we theoretically show that the higher harmonic gamma-ray produced by nonlinear inverse Thomson scattering of circularly polarized light is a gamma-ray vortex, which means that it possesses a helical wave front and carries orbital angular momentum. Our work explains a recent experimental result regarding nonlinear inverse Thomson scattering that clearly shows an annular intensity distribution as a remarkable feature of a vortex beam. Our work implies that gamma-ray vortices should be produced in various situations in astrophysics in which high-energy electrons and intense circularly polarized light fields coexist. Nonlinear inverse Thomson scattering is a promising radiation process for realizing a gamma-ray vortex source based on currently available laser and accelerator technologies, which would be an indispensable tool for exploring gamma-ray vortex science.

## Introduction

An optical vortex is an electromagnetic wave with a phase that varies azimuthally along the direction of propagation^1, 2^. When an optical vortex beam is viewed in a plane transverse to the direction of propagation, an annular intensity profile is observed due to the phase singularity at the center of the beam. An important consequence of the optical vortex is that it possesses a phase term exp(*inϕ*) that carries discrete values *nħ* of orbital angular momentum (OAM) per photon, where *n* is an integer, *ϕ* is the azimuthal angle around the central axis of the propagation, and *ħ* is the Plank constant divided by 2*π* ^1, 2^. Fundamental and applied research on optical vortices using visible wavelength lasers is ongoing^3^. However, vortex beams are not limited to the visible spectrum, but have also been generated in the ultraviolet and X-ray energy range by a helically microbunched electron beam^4^, combination of synchrotron radiation and a spiral phase plate^5, 6^, or a helical undulator^7–9^. The interaction between the optical vortex carrying OAM and matter has been investigated, e.g. optical tweezers of microscopic particles^10, 11^, OAM transfer to a valence electron^12^, new selection rules of photoionization by an ultraviolet vortex beam^13^, and a strong dichroic effect induced by a X-ray vortex^14^.

The research of optical vortices has been extended to gamma-rays with the energy of *E* > 1 MeV. Gamma-ray vortices have a potential research opportunity as a probe of the structure of hadrons^15^. If gamma-ray vortices in the energy region of 1 < *E* < 30 MeV are generated, one may study the specific interaction between gamma-ray vortices and atomic nuclei via nuclear resonance fluorescence and photonuclear reactions whose reactions depends on the total angular momentum of an incoming gamma-ray. It has been proposed on theoretical grounds that gamma-ray vortices may be generated using inverse Thomson scattering between a high energy electron and an optical vortex laser^16–18^.

In this paper, we propose for the first time an alternative method to generate a gamma-ray vortex using nonlinear inverse Thomson scattering (NITS) of a high energy electron and an extremely intense circularly polarized laser. Inverse Thomson scattering is a process by which a high-energy electron converts a low energy photon to high-energy one^19^. Inverse Thomson scattered gamma-ray beams are available at many accelerator facilities around the world for the study of nuclear physics and various applications^20–26^. In many cases, a high power laser is used to generate an intense gamma-ray beam. When the peak power of a laser is sufficiently intense, a nonlinear effect of inverse Thomson scattering occurs. In the classical picture of NITS^27^, the transverse electron motion is induced by the intense laser field and higher harmonic photons are produced. Progress in laser physics has enabled the observation of NITS in the laboratory^28, 29^.

We theoretically investigate the electric field of the gamma-ray emitted from a relativistic electron inside the intense circularly polarized laser. We show that the *n*-th harmonic gamma-rays possess the phase term exp{*i*(*n* − 1)*ϕ*} and carry (*n* − 1)*ħ* OAM. In our method, the circularly polarized laser is crucial because the phase term arises from the transverse helical motion of the electron inside the circularly polarized laser field. A recent measurement of the annular profile of second harmonic X-rays of NITS using a circularly polarized laser^29^ strongly supports our theoretical prediction.

## Methods–Derivation of theoretical equations

### An electric field of gamma-rays emitted by NITS

**Figure 1:**
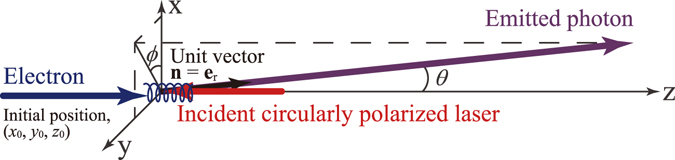
Coordinate system used in the calculation.

We treat NITS in the context of classical electrodynamics as in the previous work^27^. Here, we assume that an intense circularly polarized laser is propagating along the −*z* direction. A coordinate system used in the calculation is shown in Fig. 1. Electron orbits, **r**_*e*_ = (*x*_*e*_, *y*_*e*_, *z*_*e*_), inside a circularly polarized laser field are helical, and are given by Eqs (19a–19c) in the ref. 27

1 $${x}_{e}(\eta )={x}_{0}+{r}_{1}\,\sin \,{k}_{0}\eta ,$$

2 $${y}_{e}(\eta )={y}_{0}-{r}_{1}\,\cos \,{k}_{0}\eta ,$$

3 $${z}_{e}(\eta )={z}_{0}+{\beta }_{1}\eta ,$$

with an independent variable *η* = *z*_*e*_ + *ct*. Here, *c* is the speed of light, *t* is time, *x*_0_, *y*_0_, and *z*_0_ are the initial position of the electron along the *x*, *y*, and *z*-axis, respectively, and *r*_1_ is the radius of the helical motion given by

4 $${r}_{1}=\frac{{a}_{0}}{\sqrt{2}{\gamma }_{0}(1+{\beta }_{0}){k}_{0}}.$$

*β*_1_ is given by

5 $${\beta }_{1}=\frac{1}{2}(1-\frac{1+{a}_{0}^{2}/2}{{\gamma }_{0}^{2}{(1+{\beta }_{0})}^{2}}),$$

and *β*_1_ *c*/(1 − *β*_1_) is the drift velocity of the electron along the *z*-axis. Here, *k*_0_ = 2*π*/*λ*_0_ is the wave number of the laser field (where *λ*_0_ is the wavelength of the laser), *a*_0_ is the laser strength parameter described as ${a}_{0}=0.85\times {10}^{-9}{\lambda }_{0}(\mu {\rm{m}}){I}_{0}^{1/2}({\rm{W}}/{{\rm{cm}}}^{2})$, *I*_0_ is the intensity of the laser, *γ*_0_ and *β*_0_ = *v*_0_/*c* are the Lorentz factor and the normalized velocity of the initial electron (where *v*_0_ is the electron velocity), respectively. Equations (1–3) correspond to the case in which the electron moves counterclockwise when the observer is facing the oncoming electron, which we call positive helicity.

The Fourier component of the electric field emitted by a single electron in an arbitrary orbit and normalized velocity can be calculated from the Lienard–Wiechert potentials (section 14.5 in the ref. 30):

6 $${\bf{E}}=-i\sqrt{\frac{{e}^{2}{k}^{2}}{32{\pi }^{3}{\varepsilon }_{0}^{2}}}\frac{{e}^{ikR}}{R}{\int }_{-\infty }^{\infty }{\rm{d}}t\{{\bf{n}}\times ({\bf{n}}\times \beta )\}{e}^{i\omega \{t-\frac{{\bf{n}}\cdot {{\bf{r}}}_{e}(t)}{c}\}},$$

where *e* is the elementary charge, *ω* and *k* = *ω*/*c* are the angular frequency and the wave number of the emitted photon, respectively, *ε*_0_ is the permittivity of vacuum, *R* is the distance from the origin to the observation point, **n** is a unit vector pointing from the origin to the observation point, *β* = *v*/*c* (where *v* is the electron velocity vector inside the laser field), and **r**_*e*_ is the electron orbit described in Eqs (1–3). Equation (6) is calculated in the spherical coordinates system (*r*, *θ*, *ϕ*) and unit vectors (**e**_*r*_, **e**_*θ*_, **e**_*ϕ*_) by using following relations^27^:

7 $$\begin{array}{rcl}{\bf{n}}\times ({\bf{n}}\times \beta ) & = & -\,({\beta }_{x}\,\cos \,\theta \,\cos \,\varphi +{\beta }_{y}\,\cos \,\theta \,\sin \,\varphi -{\beta }_{z}\,\sin \,\theta ){{\bf{e}}}_{\theta }\\  &  & +({\beta }_{x}\,\sin \,\varphi -{\beta }_{y}\,\cos \,\varphi ){{\bf{e}}}_{\varphi },\end{array}$$

8 $$\beta =\frac{1}{c}\frac{{\rm{d}}{{\bf{r}}}_{e}}{{\rm{d}}t}=\frac{1}{c}(\frac{{\rm{d}}{z}_{e}}{{\rm{d}}t}+c)\frac{{\rm{d}}{{\bf{r}}}_{e}}{{\rm{d}}\eta },$$

9 $$\psi \equiv \omega (t-\frac{{\bf{n}}\cdot {{\bf{r}}}_{e}(t)}{c})=k(\eta -{z}_{e})-k({x}_{e}{\rm{\sin }}\theta \,{\rm{\cos }}\varphi +{y}_{e}{\rm{\sin }}\theta \,{\rm{\sin }}\varphi +{z}_{e}{\rm{\cos }}\theta \mathrm{).}$$

Each component of the electric field in the spherical coordinate system can be expressed as follows:

10 $${E}_{\theta }=i\sqrt{\frac{{e}^{2}{k}^{2}}{32{\pi }^{3}{\varepsilon }_{0}^{2}{c}^{2}}}\frac{{e}^{ikR}}{R}{\int }_{-{\eta }_{0}}^{{\eta }_{0}}{\rm{d}}\eta (\frac{{\rm{d}}{x}_{e}}{{\rm{d}}\eta }\,\cos \,\theta \,\cos \,\varphi +\frac{{\rm{d}}{y}_{e}}{{\rm{d}}\eta }\,\cos \,\theta \,\sin \,\varphi -\frac{{\rm{d}}{z}_{e}}{{\rm{d}}\eta }\,\sin \,\theta ){e}^{i\psi },$$

11 $${E}_{\varphi }=-i\sqrt{\frac{{e}^{2}{k}^{2}}{32{\pi }^{3}{\varepsilon }_{0}^{2}{c}^{2}}}\frac{{e}^{ikR}}{R}{\int }_{-{\eta }_{0}}^{{\eta }_{0}}{\rm{d}}\eta (\frac{{\rm{d}}{x}_{e}}{{\rm{d}}\eta }\,\sin \,\varphi -\frac{{\rm{d}}{y}_{e}}{{\rm{d}}\eta }\,\cos \,\varphi ){e}^{i\psi },$$

where, *η*_0_ = *N*_0_ *λ*_0_/2 (where *N*_0_ is the number of the periods of the laser field interacting with the electron), which corresponds to half of the laser pulse length.

The electric fields of Eqs (10) and (11) can be calculated by inserting Eqs (1–3), and using the following relations:

12 $$\begin{array}{rcl}\psi  & = & -k\{{x}_{0}\,\sin \,\theta \,\cos \,\varphi +{y}_{0}\,\sin \,\theta \,\sin \,\varphi +{z}_{0}(1+\cos \,\theta )\}\\  &  & +k\eta \{1-{\beta }_{1}(1+\,\cos \,\theta )\}-k{r}_{1}\,\sin \,\theta \,\sin ({k}_{0}\eta -\varphi )\\  & = & {\psi }_{0}+k\eta \{1-{\beta }_{1}(1+\,\cos \,\theta )\}-p\,\sin ({k}_{0}\eta -\varphi ),\end{array}$$

13 $${\psi }_{0}\equiv -k\{{x}_{0}\,\sin \,\theta \,\cos \,\phi +{y}_{0}\,\sin \,\theta \,\sin \,\varphi +{z}_{0}\mathrm{(1}+\,\cos \,\theta )\},$$

14 $$p\equiv k{r}_{1}\,\sin \,\theta ,$$

15 $$\frac{{\rm{d}}{x}_{e}}{{\rm{d}}\eta }\,\cos \,\theta \,\cos \,\varphi +\frac{{\rm{d}}{y}_{e}}{{\rm{d}}\eta }\,\cos \,\theta \,\sin \,\varphi -\frac{{\rm{d}}{z}_{e}}{{\rm{d}}\eta }\,\sin \,\theta ={k}_{0}{r}_{1}\,\cos \,\theta \,\cos ({k}_{0}\eta -\varphi )-{\beta }_{1}\,\sin \,\theta ,$$

16 $$\frac{{\rm{d}}{x}_{e}}{{\rm{d}}\eta }\,\sin \,\varphi -\frac{{\rm{d}}{y}_{e}}{{\rm{d}}\eta }\,\cos \,\varphi ={k}_{0}{r}_{1}\,\sin ({k}_{0}\eta -\varphi ),$$

and formulas:

17 $${e}^{-ip\sin \sigma }=\sum _{n=-\infty }^{\infty }{J}_{n}(p){e}^{-in\sigma },$$

18 $$\cos \,\sigma {e}^{-ip\sin \sigma }=\sum _{n=-\infty }^{\infty }\frac{n}{p}{J}_{n}(p){e}^{-in\sigma },$$

19 $$\sin \,\sigma {e}^{-ip\sin \sigma }=i\sum _{n=-\infty }^{\infty }{J}_{n}^{^{\prime} }(p){e}^{-in\sigma }\mathrm{.}$$

Here, *σ* = *k*_0_ *η* − *ϕ*, *J*_*n*_(*p*) and ${J}_{n}^{^{\prime} }(p)$ are the Bessel function of the first kind and its derivative, respectively. The result will be

20 $$\begin{array}{rcl}{E}_{\theta } & = & \sum _{n=1}^{\infty }i\sqrt{\frac{{e}^{2}{k}^{2}{\lambda }_{0}^{2}{N}_{0}^{2}}{32{\pi }^{3}{\varepsilon }_{0}^{2}{c}^{2}}}\frac{\sin (\overline{k}{\eta }_{0})}{\overline{k}{\eta }_{0}}(\frac{n{k}_{0}\,\cos \,\theta }{k\,\sin \,\theta }-{\beta }_{1}\,\sin \,\theta )\,{J}_{n}(p)\frac{{e}^{i{\psi }_{0}+ikR+in\varphi }}{R}\\  & \equiv  & \sum _{n=1}^{\infty }i{C}_{\theta }\frac{{e}^{i{\psi }_{0}+ikR+in\varphi }}{R},\end{array}$$

21 $$\begin{array}{rcl}{E}_{\varphi } & = & \sum _{n=1}^{\infty }(-)\sqrt{\frac{{e}^{2}{k}^{2}{\lambda }_{0}^{2}{N}_{0}^{2}}{32{\pi }^{3}{\varepsilon }_{0}^{2}{c}^{2}}}\frac{\sin (\overline{k}{\eta }_{0})}{\overline{k}{\eta }_{0}}{r}_{1}{k}_{0}{J}_{n}^{^{\prime} }(p)\frac{{e}^{i{\psi }_{0}+ikR+in\varphi }}{R}\\  & \equiv  & \sum _{n=1}^{\infty }(-){C}_{\varphi }\frac{{e}^{i{\psi }_{0}+ikR+in\varphi }}{R},\end{array}$$

where,

22 $$\overline{k}=k\{1-{\beta }_{1}(1+\,\cos \,\theta )\}-n{k}_{0},$$

and *n* is the integer and related to the harmonic number of the emitted gamma-ray (*n* ≥ 1).

To show the phase structure in the transverse plane, we express the electric field in the orthogonal coordinate system, as expressed below:

23 $${E}_{x}={E}_{\theta }\,\cos \,\theta \,\cos \,\varphi -{E}_{\varphi }\,\sin \,\varphi ,$$

24 $${E}_{y}={E}_{\theta }\,\cos \,\theta \,\sin \,\varphi +{E}_{\varphi }\,\cos \,\varphi ,$$

25 $${E}_{z}=-{E}_{\theta }\,\sin \,\theta .$$

Thus, the electric field is

26 $$\begin{array}{rcl}{\bf{E}} & = & {E}_{x}{{\bf{e}}}_{x}+{E}_{y}{{\bf{e}}}_{y}+{E}_{z}{{\bf{e}}}_{z}\\  & = & \frac{{E}_{x}-i{E}_{y}}{\sqrt{2}}{{\bf{e}}}_{+}+\frac{{E}_{x}+i{E}_{y}}{\sqrt{2}}{{\bf{e}}}_{-}+{E}_{z}{{\bf{e}}}_{z}\\  & = & \sum _{n=1}^{\infty }\{\frac{i({C}_{\theta }\,\cos \,\theta +{C}_{\varphi })}{\sqrt{2}}\frac{{e}^{i{\psi }_{0}+ikR+i(n-1)\varphi }}{R}{{\bf{e}}}_{+}+\frac{i({C}_{\theta }\,\cos \,\theta -{C}_{\varphi })}{\sqrt{2}}\frac{{e}^{i{\psi }_{0}+ikR+i(n+1)\varphi }}{R}{{\bf{e}}}_{-}\\  &  & -i{C}_{\theta }\,\sin \,\theta \frac{{e}^{i{\psi }_{0}+ikR+in\varphi }}{R}{{\bf{e}}}_{z}\}.\end{array}$$

Here, the electric field is expressed by the complex orthogonal unit vectors ${{\bf{e}}}_{\pm }\equiv ({{\bf{e}}}_{x}\pm i{{\bf{e}}}_{y})/\sqrt{2}$ whose helicities of the circularly polarized electric fields of the gamma-rays are positive and negative, respectively. In the paraxial approximation ($\theta \ll 1$) the longitudinal component of the electric field along the *z*-axis is much smaller than the transverse component. With the assumption of (*x*_0_, *y*_0_, *z*_0_) = (0, 0, 0) and $R\simeq z$ (where *z* is the distance along the *z*-axis from the origin to the observation point), the electric field can be expressed as follows:

27 $${\bf{E}}=\sum _{n=1}^{\infty }\{\frac{i({C}_{\theta }+{C}_{\varphi })}{\sqrt{2}}\frac{{e}^{ikz+i(n-1)\varphi }}{z}{{\bf{e}}}_{+}+\frac{i({C}_{\theta }-{C}_{\varphi })}{\sqrt{2}}\frac{{e}^{ikz+i(n+1)\varphi }}{z}{{\bf{e}}}_{-}\}.$$

### Degree of circular polarization of gamma-rays

The degree of circular polarization of the gamma-rays can be represented by the Stokes parameter, *S*_3_/*S*_0_, which is expressed as (section 7.2 in the ref. 30)

28 $$\begin{array}{rcl}\frac{{S}_{3}}{{S}_{0}} & = & \frac{{({C}_{\theta }\cos \theta +{C}_{\varphi })}^{2}-{({C}_{\theta }\cos \theta -{C}_{\varphi })}^{2}}{{({C}_{\theta }\cos \theta +{C}_{\varphi })}^{2}+{({C}_{\theta }\cos \theta -{C}_{\varphi })}^{2}}=\frac{2{C}_{\theta }{C}_{\varphi }\,\cos \,\theta }{{C}_{\theta }^{2}{\cos }^{2}\theta +{C}_{\varphi }^{2}}\\  & = & \frac{2(\frac{n{k}_{0}\,\cos \,\theta }{k\,\sin \,\theta }-{\beta }_{1}\,\sin \,\theta ){r}_{1}{k}_{0}{J}_{n}(p){J}_{n}^{^{\prime} }(p)\cos \,\theta }{{\{(\frac{n{k}_{0}\cos \theta }{k\sin \theta }-{\beta }_{1}\sin \theta ){J}_{n}(p)\cos \theta \}}^{2}+{\{{r}_{1}{k}_{0}{J}_{n}^{^{\prime} }(p)\}}^{2}}.\end{array}$$

When the following inequality is satisfied,

29 $$\frac{p}{2} < \sqrt{n},$$

the Bessel function of the first kind and its derivative can be expressed as

30 $${J}_{n}(p)\simeq \frac{1}{n!}{(\frac{p}{2})}^{n},$$

31 $${J}_{n}^{^{\prime} }(p)=\frac{1}{2}\{{J}_{n-1}(p)-{J}_{n+1}(p)\}\simeq \frac{1}{2(n-1)!}{(\frac{p}{2})}^{n-1}.$$

Therefore, in the paraxial approximation ($\theta \ll 1$) Eq. (28) will be

32 $$\frac{{S}_{3}}{{S}_{0}}=\frac{2(\frac{n{k}_{0}}{k\theta }-{\beta }_{1}\theta )\frac{p}{{r}_{1}{k}_{0}}n!(n-1)!}{{(\frac{n{k}_{0}}{k\theta }-{\beta }_{1}\theta )}^{2}{(\frac{p}{{r}_{1}{k}_{0}})}^{2}(n-1){!}^{2}+n{!}^{2}}=\frac{2\alpha nn!(n-1)!}{{\alpha }^{2}{n}^{2}(n-1){!}^{2}+n{!}^{2}}=\frac{2\alpha }{{\alpha }^{2}+1}.$$

Here,

33 $$\alpha \equiv 1-\frac{4{\gamma }_{0}^{2}{\theta }^{2}}{2{\gamma }_{0}^{2}{\theta }^{2}+2+{a}_{0}^{2}}.$$

Therefore, when Eq. (29) is satisfied, the Stokes parameter does not depend on the harmonic number, *n*.

### Energy, transverse energy distribution, and the number of photons of gamma-ray vortices

Equations (20) and (21) indicate that the function, $\sin (\overline{k}{\eta }_{0})/(\bar{k}{\eta }_{0})$, is sharply peaked at wave number given by $\overline{k}=0$ and the energy bandwidth of *n*-th harmonic gamma-rays is given by Δ*ω*/*ω*_*n*_ = 1/(*nN*_0_)^27^. The energy at the maximum intensity of the *n*-th harmonic gamma-ray can be calculated by Eq. (22), when $\overline{k}=0$,

34 $${\omega }_{n}=\frac{n{\omega }_{0}}{1-{\beta }_{1}(1+\,\cos \,\theta )}=\frac{8n{\gamma }_{0}^{2}{\omega }_{0}}{2{\gamma }_{0}^{2}{\theta }^{2}+2+{a}_{0}^{2}}.$$

Here, *ω*_0_(=*k*_0_ *c*) is the angular frequency of the initial circularly polarized laser.

The radiation energy per angular frequency and solid angle is expressed as (section 14.5 in the ref. 30)

35 $$\begin{array}{rcl}\frac{{d}^{2}I}{d\omega d{\rm{\Omega }}} & = & 2{\varepsilon }_{0}c{R}^{2}{|{\bf{E}}|}^{2}\\  & = & \frac{{e}^{2}{k}^{2}{\lambda }_{0}^{2}{N}_{0}^{2}}{16{\pi }^{3}{\varepsilon }_{0}c}[{\{\sum _{n=1}^{\infty }\frac{\sin \overline{k}{\eta }_{0}}{\overline{k}{\eta }_{0}}\frac{n{k}_{0}\cos \theta -k{\beta }_{1}{\sin }^{2}\theta }{k\sin \theta }{J}_{n}(p)\}}^{2}\\  &  & +{\{\sum _{n=1}^{\infty }\frac{\sin \overline{k}{\eta }_{0}}{\overline{k}{\eta }_{0}}{r}_{1}{k}_{0}{J}_{n}^{^{\prime} }(p)\}}^{2}],\end{array}$$

here, dΩ = d*ϕ*d*θ *sin*θ*. The number of *n*-th harmonic photons emitted per second toward the scattering angle between *θ*_1_ and *θ*_2_ can be approximately calculated from Eq. (35), when $\overline{k}=0$,

36 $$\begin{array}{rcl}N & = & \frac{{N}_{e}{\rm{\Delta }}\omega }{\hslash {\omega }_{n}}{\int }_{0}^{2\pi }{\rm{d}}\varphi {\int }_{{\theta }_{1}}^{{\theta }_{2}}{\rm{d}}\theta \,\sin \,\theta \frac{{{\rm{d}}}^{2}I}{{\rm{d}}\omega {\rm{d}}{\rm{\Omega }}}\\  & = & \frac{{N}_{e}}{n\hslash {N}_{0}}{\int }_{0}^{2\pi }{\rm{d}}\varphi {\int }_{{\theta }_{1}}^{{\theta }_{2}}{\rm{d}}\theta \,\sin \,\theta \frac{{{\rm{d}}}^{2}I}{{\rm{d}}\omega {\rm{d}}{\rm{\Omega }}},\end{array}$$

where *N*_*e*_ is the number of the electrons interacting with the laser per second. Here, we take into account the bandwidth of scattered photons arising from the finite laser pulse length, Δ*ω*/*ω*_*n*_ = 1/(*nN*_0_)^27^. Even if a single cycle laser is used (*N*_0_ = 1), the gamma-ray vortices may be generated. However, the number of photons is proportional to *N*_0_ and the bandwidth of gamma-ray energy is inversely proportional to *N*_0_. Therefore, a long cycle laser is more useful than a single cycle laser for a nuclear physics experiment, which desires an intense gamma-ray source with a narrow energy bandwidth.

## Results

### Spatial energy distribution of gamma-ray vortices

**Figure 2:**
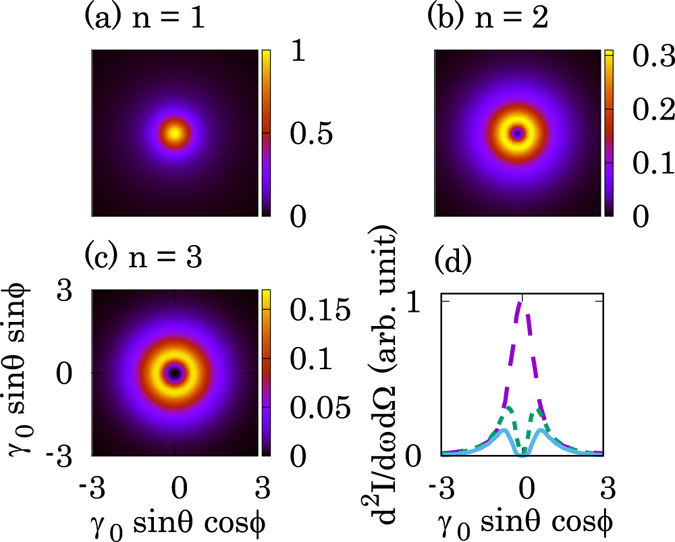
Calculated spatial distribution of gamma rays generated by NITS with a circularly polarized laser. Spatial intensity distributions of the (**a**) first, (**b**) second, and (**c**) third harmonics, calculated by Eq. (35) and normalized by the maximum value of the first harmonic. (**d**) Line intensity distribution along the x-axis for each harmonic number. The lines indicate the following: long dashed line, *n* = 1; short dashed line, *n* = 2; and solid line, *n* = 3. The other parameters are *γ*_0_ = 2000, *λ*_0_ = 1.0 *μ*m, *a*_0_ = 1.0, and *N*_0_ = 500. An annular shape and zero intensity at the z-axis appear only for the higher harmonics. These features are consistent with the characteristics of vortex beams.

Figure 2 shows the spatial energy distribution in the transverse plane for each harmonic number calculated using Eq. (35). Here, we used following parameters: *γ*_0_ = 2000, *λ*_0_ = 1.0 *μ*m, *a*_0_ = 1.0, and *N*_0_ = 500. Most photons generated by NITS are concentrated near the central axis. A single maximum in the distribution is observed in the fundamental harmonic gamma-rays (Fig. 2(a)). An annular shape and with zero intensity at the z-axis occurs only for the higher harmonics (Fig. 2(b,c)). We stress that this feature in the higher harmonics is a typical characteristics of a vortex beam.

### Relation between the polarization state and OAM

Equation (27) shows that the electric field of the gamma-ray is an elliptically polarized wave, which can be decomposed into circularly polarized components with positive and negative helicities. More importantly, Eq. (27) shows that the phase term is characterized by the unit vector representing the helicity of circularly polarized gamma-rays. The higher harmonics (*n* ≥ 2) of the positive helicity component possess a helical phase structure represented by the phase term exp{*i*(*n* − 1)*ϕ*}, and all harmonics of the negative helicity component possess the phase term exp{*i*(*n* + 1)*ϕ*}.

**Figure 3:**
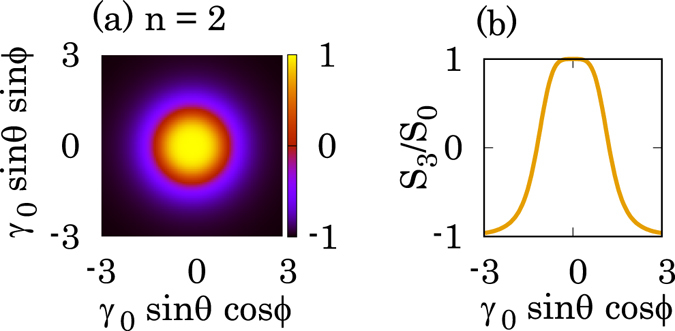
Calculated Stokes parameters of circularly polarized gamma rays generated by NITS. The (**a**) spatial and (**b**) line distributions of the degree of the circular polarization in the x–y plane calculated by Eq. (28). The parameters are *γ*_0_ = 2000, *λ*_0_ = 1.0 *μ*m, *a*_0_ = 1.0, *N*_0_ = 500, and *n* = 2. *S*_3_/*S*_0_ = 1(−1) means 100% circular polarization with positive (negative) helicity. In the scattering angle of *θ* < 0.6/*γ*_0_ and 2.40/*γ*_0_ < *θ*, the degree of the circular polarization of the gamma-rays are more than 90% circular polarization with positive and negative helicity, respectively.

The degree of circular polarization of the gamma-rays can be represented by the Stokes parameter (section 7.2 in the ref. 30). Figure 3 shows the spatial distribution of the Stokes parameter calculated by Eq. (28). We used the parameters of *γ*_0_ = 2000, *λ*_0_ = 1.0 *μ*m, *a*_0_ = 1.0, *N*_0_ = 500, and *n* = 2. Equation (29) is valid when the following conditions are satisfied, 0 ≤ *γ*_0_ *θ* ≤ 3, 0 ≤ *a*_0_ ≤ 1, and *n* ≤ 10, respectively. Therefore, the contour of the Stokes parameter is similar up to the tenth harmonics. The Stokes parameters of *S*_3_/*S*_0_ = 1, −1, and 0 indicate 100% circular polarization with positive and negative helicity, and 100% linear polarization, respectively. We found that in the scattering angle of *θ* < 0.6/*γ*_0_, the degree of the circular polarization of the gamma-rays is more than 90% of circular polarization with positive helicity, which coincides with the electron motion. The degree of circular polarization decreases as the scattering angle increases. The polarization changes from circular to linear polarization near the angle of *θ* = 1.2/*γ*_0_. The polarization again changes to more than 90% of circular polarization with negative helicity at a greater scattering angle of 2.40/*γ*_0_ < *θ*. As a result, only higher harmonic gamma-rays emitted in the scattering angle of *θ* < 0.6/*γ*_0_ should carry (*n* − 1)*ħ* OAM. The helicity of the circular polarization becomes negative at large scattering angles. Gamma-rays emitted in the scattering angle of 2.40/*γ*_0_ < *θ* carry (*n* + 1)*ħ* OAM.

The generation of vortex beams by NITS is physically similar to helical undulator radiation, where the trajectory of an electron inside a magnetic field is helical. The generation of vortex beams from helical undulators was theoretically proposed^7^ and experimentally verified later^9^. The phase term, exp{*i*(*n* − 1)*ϕ*}, can be seen in the complex amplitude of an emitted photon and the annular intensity distribution was also observed only in the higher harmonics (Eq. (6) and Fig. 2 in the ref. 7). Our theoretical results are consistent with their results.

### Expected intensity and energy

**Figure 4:**
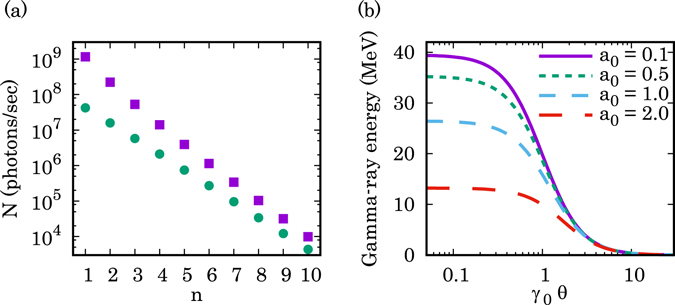
(**a**) Number of photons per unit time at each harmonic calculated using Eq. (36). The integration range of the scattering angle of each symbol is as follows: square, *θ*_1_ = 0.003/*γ*_0_ and *θ*_2_ = 0.6/*γ*_0_; circle, *θ*_1_ = 2.40/*γ*_0_ and *θ*_2_ = 2.47/*γ*_0_. These regions correspond to the more than 90% of circular polarization with positive helicity carrying OAM and more than 90% of circular polarization with negative helicity carrying OAM, respectively. The calculation parameters are as follows: *γ*_0_ = 2000, *λ*_0_ = 1.0 *μ*m, *a*_0_ = 1.0, *N*_0_ = 500, and *N*_*e*_ = 10^9^ electrons/s. (**b**) Energy of NITS gamma-rays (*n* = 2) calculated by Eq. (34). The laser strength parameters are as follows: *a*_0_ = 0.1 (solid line), *a*_0_ = 0.5 (short dashed line), *a*_0_ = 1.0 (middle dashed line), and *a*_0_ = 2.0 (long dashed line). Other calculation parameters are *γ*_0_ = 2000 and *λ*_0_ = 1.0 *μ*m.

We estimate the expected number of photons and their energy for a gamma-ray vortex source based on NITS using currently available laser and accelerator technology. The calculated results for the number of photons and the energy of NITS gamma-ray vortices are shown in Fig. 4(a,b), respectively. The calculation parameters are: *γ*_0_ = 2000, *λ*_0_ = 1.0 *μ*m, *a*_0_ = 1.0, *N*_0_ = 500, and *N*_*e*_ = 10^9^ electrons/s. These parameters are readily available at modern mid-energy scale accelerator facilities with high power lasers. It is possible to obtain 10^9^ photons/s at the fundamental component, with more than 90% circular polarization of positive helicity and with energies of 11–13 MeV, but not carrying any OAM. In contrast, the second harmonic contains 2 × 10^8^ photons/s with energies of 21–26 MeV (Fig. 4(b)) and carries 1*ħ* OAM. At the large angles of 2.40/*γ*_0_ < *θ*, these photons predominantly possess more than 90% of circular polarization with negative helicity, and carry (*n* + 1)*ħ* OAM; however, the number of the photons is smaller by one order of magnitude than that with positive helicity up to the third harmonics.

It is well known that in inverse Thomson scattering, the energy of the emitted gamma-rays depends on the electron beam energy, the wavelength of the laser, and the scattering angle. However, in the case of NITS, it also depends on the laser strength parameter, *a*_0_. The energy of the second harmonic gamma-ray as a function of the scattering angle for each laser strength parameter can be calculated by Eq. (34) and is shown in Fig. 4(b). The gamma-ray energy decreases as the laser strength parameter in the region *a*_0_ > 1 increases due to a decrease in the longitudinal electron velocity and a growth of the transverse helical motion. The energy of the emitted gamma rays can be tuned by changing the electron beam energy and the wavelength of the laser.

## Discussion

Thomson scattering theory is valid when the scattered photon energy is much lower than the electron energy, i.e., $\hslash {\omega }_{n}\ll {\gamma }_{0}{m}_{e}{c}^{2}$ as described in the ref. 27, where *m*_*e*_ is the electron energy at rest. Under the condition with *λ*_0_ = 1 *μ*m, *a*_0_ = 1, *n* = 2, and *θ* = 0, this implies ${\gamma }_{0}\ll 5\times {10}^{4}$ and *ħω*_2_ ≪ 25 GeV. Therefore, our calculation result is valid for gamma-ray vortex generation in the MeV energy range.

It was proposed that gamma ray vortices with energies greater than MeV could be generated, using inverse Thomson scattering between an electron and an optical vortex laser^16^. In this method, the OAM value of the vortex laser is considered to be conserved in the small scattering angle of the order of $1/{\gamma }_{0}^{2}$ as described in the ref. 16. Only a small fraction of the gamma-ray photons scatter into this small angle and many others scatter in the angle of 1/*γ*_0_ in inverse Thomson scattering. In contrast, in the our proposed method, the gamma ray vortices can be generated into the angle in the order of 1/*γ*_0_. Therefore it seems that the generation efficiency of the present method is approximately higher by the factor of *γ*_0_ than that of the vortex laser method.

**Figure 5:**
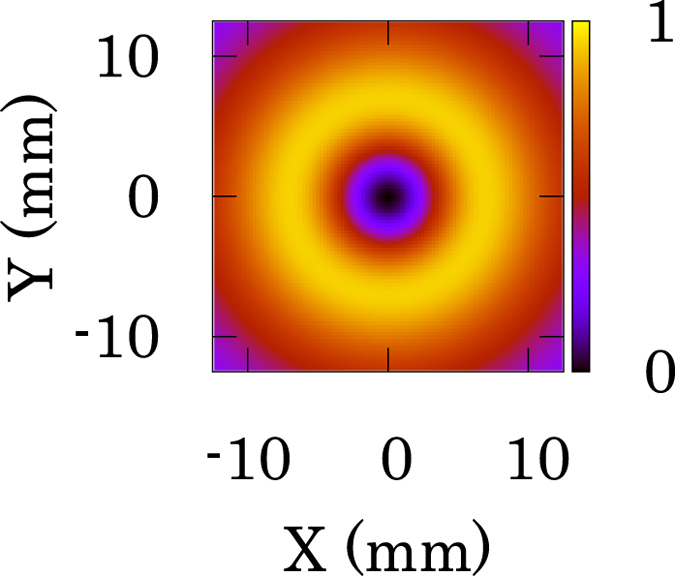
Spatial distribution of the second harmonic X-rays in the case of Brookhaven National Laboratory calculated by Eq. (35). The parameters are *γ*_0_ = 128, *λ*_0_ = 10.6 *μ*m, *a*_0_ = 0.6, *n* = 2, and *z* = 1.85 m.

An important result in the present study is that the annular intensity distribution of the higher harmonic gamma-rays is generated by NITS as shown in Fig. 2. NITS using a relativistic electron beam and a laser has been experimentally investigated at accelerator facilities^28, 29^. Recently, NITS was demonstrated using a circularly polarized laser with a laser strength parameter of *a*_0_ = 0.6 at the Brookhaven National Laboratory (BNL)^29^. The results showed an annular shape of the intensity distribution for the second harmonics of scattered X-rays with an energy of 13 keV (Fig. 8 in the ref. 29), although it was stated that this shape originated from a helical motion of the electron. However, in the present study, we can reproduce the annular shape of the second harmonic X-rays by using parameters of the electron accelerator and the laser at BNL as shown in Fig. 5. This indicates that the measured annular profile resulted from X-ray vortices generated by NITS.

### Gamma-ray vortices for the study of nuclear physics

Photons with non-zero OAM and energies larger than 1 MeV will open a new frontier in nuclear physics and nuclear astrophysics. The interaction between atomic nuclei and photons with energies of 1 < *E* < 30 MeV are dominated by nuclear resonances fluorescence and photo nuclear reactions. These reactions can be understood by a two-step model. First, an incident photon is absorbed by a nucleus and thereby the nucleus is exited. If the excitation energy is lower than particle separation threshold, the excited nucleus subsequently decays out through nuclear resonances fluorescence with the emission of a gamma-ray or a cascade of gamma-rays. If the excited energy is higher than the particle threshold, the nucleus can decay out to a residual nucleus with the emission of a particle such as neutron or proton (photo nuclear reaction). The spin and parity of the excited nucleus populated by the absorption of a photon is limited by the conservation laws of angular momentum^31^, namely, |*I*_*i*_ − *I*_*p*_| ≤ *I*_*f*_ ≤ |*I*_*i*_ + *I*_*p*_|, where *I*_*i*_ and *I*_*f*_ are the spin of the initial state and the exited state in the nucleus, respectively, and *I*_*p*_ is the total angular momentum of the incident photon. It is well known that, for the photon-nucleus interactions, giant dipole resonances (GDRs) play the dominant role at 10–30 MeV. The GDRs are commonly observed in most nuclei except for proton. Because the GDRs should be excited by electric-dipole (E1) transitions, the spin and parity of GDRs in *J* ^*π*^ = 0^+^ even-even nuclei is *J* ^*π*^ = 1^−^. If a gamma-ray vortex whose total angular momentum is higher than or equal to 2*ħ* induces on an *J* ^*π*^ = 0^+^ even-even nucleus, the GDR excitation should be forbidden because of the conservation laws of angular momentum as discussed above. Therefore, the photonuclear reaction cross section should be drastically changed at least in the GDR region. Finally, we would like to point out that gamma-rays play also an important role in stellar nucleosynthesis, such as the *γ*-process in supernova explosions, in which some rare isotopes are produced by successive photodisintegration reactions^32^. High-energy gamma ray vortices may be generated in stellar environments in which high-energy electrons and intense circularly polarized electromagnetic fields coexist, for example, the vicinity of magnetized neutron stars^33–35^ or magnetohydrodynamic jet supernova explosions^36^. If gamma-ray vortex will available in laboratories, it will be a new probe to study nuclear physics and nuclear astrophysics.
